# Photo2Video: Semantic-Aware Deep Learning-Based Video Generation from Still Content

**DOI:** 10.3390/jimaging8030068

**Published:** 2022-03-10

**Authors:** Paula Viana, Maria Teresa Andrade, Pedro Carvalho, Luis Vilaça, Inês N. Teixeira, Tiago Costa, Pieter Jonker

**Affiliations:** 1 INESC TEC, 4200-465 Porto, Portugal or mandrade@fe.up.pt (M.T.A.); pedro.carvalho@inesctec.pt (P.C.); luis.m.salgado@inesctec.pt (L.V.); ines.f.teixeira@inesctec.pt (I.N.T.); tiago.a.costa@inesctec.pt (T.C.); 2 School of Engineering, Polytechnic of Porto, 4200-072 Porto, Portugal; 3 Faculty of Engineering, University of Porto, 4200-465 Porto, Portugal; 4 QdepQ Systems, 2611NP Delft, The Netherlands; p.p.jonker@qdepq.com; 5 TU Delft Robotics Institute, 2600AA Delft, The Netherlands

**Keywords:** deep learning, semantic awareness, context awareness, RoI, automated content creation, storytelling

## Abstract

Applying machine learning (ML), and especially deep learning, to understand visual content is becoming common practice in many application areas. However, little attention has been given to its use within the multimedia creative domain. It is true that ML is already popular for content creation, but the progress achieved so far addresses essentially textual content or the identification and selection of specific types of content. A wealth of possibilities are yet to be explored by bringing the use of ML into the multimedia creative process, allowing the knowledge inferred by the former to influence automatically how new multimedia content is created. The work presented in this article provides contributions in three distinct ways towards this goal: firstly, it proposes a methodology to re-train popular neural network models in identifying new thematic concepts in static visual content and attaching meaningful annotations to the detected regions of interest; secondly, it presents varied visual digital effects and corresponding tools that can be automatically called upon to apply such effects in a previously analyzed photo; thirdly, it defines a complete automated creative workflow, from the acquisition of a photograph and corresponding contextual data, through the ML region-based annotation, to the automatic application of digital effects and generation of a semantically aware multimedia story driven by the previously derived situational and visual contextual data. Additionally, it presents a variant of this automated workflow by offering to the user the possibility of manipulating the automatic annotations in an assisted manner. The final aim is to transform a static digital photo into a short video clip, taking into account the information acquired. The final result strongly contrasts with current standard approaches of creating random movements, by implementing an intelligent content- and context-aware video.

## 1. Introduction

Multimedia content has become ubiquitous, being present in almost all aspects of our daily lives. Pictures, videos, posts, narratives, music, etc., addressing no matter what subject or topic, are all out there at the easy reach of everybody, quickly spread through social media by anyone, at any time. Such abundance is posing difficulties to professional producers, whose content is losing relevance. On the other hand, the great advances in technology, namely within the Artificial Intelligence (AI) domain, create new opportunities. Re-purposing content, innovating and publishing as quickly as possible are the keys to successfully overcoming existing barriers. This is the strategy many players have adopted already, making use of AI tools to better meet their audiences’ expectations. However, the potential benefits of such technology are still underexplored, as their use has been essentially concentrated on automatically understanding the current interests of consumers and consequently identifying and selecting specific types of content to be made available. This is known as keyword research and topic generation, so that media content can be automatically selected and published according to what customers are really interested in. Another well-covered area is the automatic generation of textual content (natural language processing and generation). Thus, the emphasis has been on automating and speeding up time-consuming and human-resource-intensive tasks, aiming for a competitive advantage in the bustling media industry landscape.

The benefits of introducing AI-based tools in the creative process itself have not yet been fully explored. We believe that, while not being able to replace the creativity and spontaneity of human artists, such tools can help in quickly obtaining more engaging and disparate content, conveying messages in cleverer ways. Automatic image analysis using AI tools can provide machine-interpreted knowledge about visual items, elaborating descriptions on the content captured in those items. When speaking about photographs, typically, content is a digital representation of a real-world event or situation. Accordingly, the use of additional contextual data characterizing the situation where and when the photo was taken is also valuable knowledge that enriches the descriptions, consequently facilitating the creation of more vivid, striking, powerful content. Our vision is that it is possible to produce automatically content-aware media clips from a single photograph by contextualizing it as much as possible, including the situation where and when the photo was taken. Such contextualization, in the form of metadata, can then be fed into intelligent creative tools which will apply cool visual effects in an automated way, obtaining contextually and semantically aware multimedia stories. The challenge of putting into life this concept and demonstrating its potential was embraced by the FotoInMotion (FiM) European consortium. The proposed solution represents a significant advance in the approaches currently used for transforming static content (photographs) into motion images (video). Instead of applying random-based movements, our work enables defining the motion based on extracted information, highlighting elements relevant for a given application scenario. Additionally, adding special effects based on the relevance of given regions of the image or on the acquiring context strongly contributes also to the final quality of the created content. In FiM, the users of the technology were active in the fields of content marketing for the fashion industry, content branding for events and content dissemination in photojournalism. Deep neural network-based models have been trained to identify concepts specific to each of the three scenarios and consequently perform region-based semantic annotation of photographs. Different types of visual dynamic effects have been identified, including motion and 3D, aiming at transforming pictures into rich video stories. We have designed mobile and desktop prototype applications incorporating such tools, implementing a creative workflow from content acquisition, through contextual data generation, to automated and/or assisted video content production. Such applications were tested by professionals in real-world conditions, demonstrating the validity of the approach.

## 2. Related Work

The use of AI-based tools to engage audiences in media content is becoming common practice. Tools like Magisto [1], Animoto [2], Flixel [3] and Facebook enable the production of low-cost videos with rudimentary text, filters and animation capabilities. However, these tools work in a rather blind way in relation to the content, applying random or pre-defined fixed animations like freezing a central object and animating a part of the photo.

The automatic creation of semantic-aware stories has been explored mainly by the Natural Language Processing (NLP) community. Examples include approaches to write stories given a title or a topic [4]; generating different story paths or plot lines from a document collection [5]; or reorganizing information to produce semantic hypertext views of a document collection [6]. Work has also been proposed to automatically generate narratives or summaries from a photo stream or album [7,8,9] and to select a representative photo [10]. Despite using contextual data, namely obtained from device sensors like GPS coordinates, as well as visual content characteristics (e.g., color histogram and identification of people), these approaches target mainly textual content generation or organization of photo assets.

On the other hand, powerful platforms such as Google Vision API [11], Microsoft Vision API [12] or Clarifai [13] are able to automatically understand the photo’s content and assign labels to the whole image, but they will not help in the creative process, nor will they provide the means for creating new multimedia content with object-based animations automatically applied to the analyzed photo.

Convolution neural networks (CNN) have gained great popularity for the recognition of concepts in visual content [14]. They implement an ML model that is able to predict a class label vector *ŷ* $\in {\left[0,1\right]}^{m}$ for an input sample x∈R, given a set of classes $C=C_{i},i=1,\ldots ,m$. The class label vector *ŷ* indicates how probable it is that the given sample *x* belongs to each of the defined classes or concepts $C_{i}$. CNNs can be used for single-target classification where classes are mutually exclusive, meaning that the input vector can belong only to one class, or for multi-target classification. The neural network model referred as “AlexNet” was the first to demonstrate the true potential of using (deep) CNNs in the computer vision domain [15]. The use of a rectified linear activation function (ReLU), as the non-linearity to substitute the up-to-then used Sigmoid and hyperbolic tangent functions which suffer from the vanishing gradients problem, was the key to its success. Nonetheless, more sophisticated activation functions have been introduced since then to overcome the bias shift problem of the rectified type of functions. Such is the case of the functions Leaky Rectified Linear Unit (LReLU), the Parametric Rectified Linear Unit (PReLU) or the Randomized Leaky Rectified Linear Unit (RReLU) [16]. Innovations within the structure of the models have also been introduced, notably with the design of the inception module, which is basically a block of parallel convolutional layers with filters of varying sizes. The model GoogLeNet [17] adopts such architecture. The Residual Network (ResNet), introduced in 2016, was able to increase the depth of the network by using residual blocks, which are sets of two convolutional layers where the outputs are combined with the inputs, establishing a shortcut connection [18]. Today, it is currently accepted that the inception and the residual models are the ones that provide better performances in computer vision tasks [19].

The work presented in this paper makes use of the Inception Resnet v2 and Resnet 101 models to automatically generate region-based semantic annotations of photographs, consequently using such metadata in an innovative way for content creation. It combines such semantic annotations with situational data to better contextualize the analyzed photo and to provide clues to intelligent editing tools on how to create engaging multimedia stories that represent in a richer way the real-world event captured initially in the photograph. A prototype system was devised to enable the implementation of such an intelligent creative workflow.

As a side-by-side result, this work offers an intuitive Web-based tool to annotate on a regional basis large image datasets, automatically and/or in an assisted manner, following former results on crowdsourced metadata [20,21,22].

## 3. Smart Video Creator System—Overall Functionality

**Figure 1:**
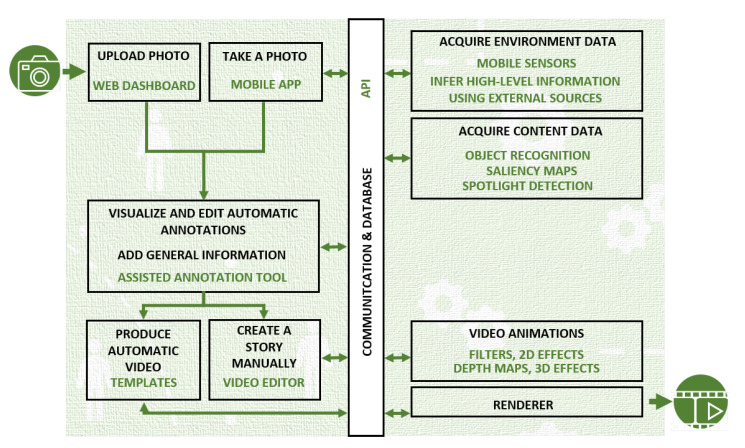
Smart video creator system.

The developed system offers two modes for the user to interact with its services: (i) a mobile app, with which the user may take a photo and seamlessly attach situational contextual data to it; and (ii) a desktop, Web-based application. Figure 1 illustrates the functionality offered by the system. In both versions, the user may upload a captured photo to the Web platform and request that semantic annotations are automatically created on a region basis. When using the mobile application to capture the photograph, contextual information will be acquired and will be uploaded to the system together with the photo. Such contextual information aims at offering a characterization of the situation when and where the photo was taken. Among other, it includes the GPS coordinates, surround sound or illumination levels. It is obtained by automatically accessing the mobile phone sensors and by processing the sensed data to infer additional knowledge, such as the name of the place or the type of sound. The automatic region-based annotation of the photograph is accomplished using not only the referred CNN models but also more traditional computer vision techniques based on feature extraction, in this case, to detect salient regions in the photo. Once this process has been performed, the user may visualize the result using a collaborative tool for assisted annotation. The user may decide to modify or adjust them or even to create new annotations. This process is done in a guided or assisted manner not only with the goal of facilitating the task of the user, but also to ensure coherence of the annotations. Finally, the user may request that the system create a multimedia animated story based on the acquired photograph, in which case 2D and 3D artistic filters and effects will be applied taking into consideration the derived metadata. Alternatively, a story can be created manually, albeit taking into account the automatic annotations.

The following hypothetical use case presents in a more concrete way how the system could be used in a realistic situation within the fashion marketing field:

“John is a professional photographer who is covering a fashion show in a small town in the north of Italy. Equipped with his smartphone and professional cameras, he decides to start taking pictures using the former, where he had previously installed the FiM app. In this way, John knows that contextual data will be automatically attached to the photos and that aspects such as the name of the place and of the event will be readily available. After uploading the initial photos to the Web platform and requesting their automatic annotation, John uses the assisted-annotation tool of the platform to visualize the produced region-based tags. He confirms that relevant objects such as the models, clothing items and accessories, as well as some perceptually relevant regions, have been detected. However, he notices some details in one of the photos that he thinks would be great to use to trigger animation and special effects. Accordingly, he adds manually bounding boxes and tags to those details, thus enriching the previously generated contextual data. Aiming for fast promotion of the event on social media, John requests the creation of a story from that photo. The creative tools are thus called upon to introduce zooming, depth-aware effects and color filters in the identified objects, navigating through the photo in a storytelling sequence.”

## 4. Semantic Information Extraction

Metadata is at the core of the automatic creative process and is captured at different stages of the workflow. Situational context data may be collected when the photograph is taken, using the mobile app. Subsequently, the context of the photo’s content is obtained using computer vision techniques to detect and tag specific objects or regions.

### 4.1. Situational Context Data

This type of contextual data is acquired by accessing sensors available on mobile devices where the FiM app is installed [23]. The use of the Intel Context Sensing package [24] enables us to infer additional knowledge from the sensed data. Examples include: Activity Recognition (e.g., “Sedentary”, “Running”), Audio Classification (e.g., “Speech”, “Music”, “Mechanical”), or Relative Location (e.g., “In”, “Out”, “Moving”, “Staying”). The system also has the possibility of using such sensed data to search for further external information through the Google Search and Google Places APIs. This may enable to associate a photo with a given place and event. For example, it may provide an indication that the photo was acquired at the place “Teatro alla Scala” during the event “Romeo e Giulietta”. These contextual data can be used in the video creation process, enabling, for instance, the addition of text and music that fit the real-world event captured in the photo.

### 4.2. Visual Feature Extraction and Classification

The automatic identification of regions of interest (RoI) within images is achieved through computer vision approaches. When using deep CNNs, specific classes of objects were defined as to cover the requirements identified for the target application scenarios (fashion, photojournalism and festivals/events): persons and genre; public personalities; clothing items and fashion accessories; symbols, such as logos and flags, etc. A total of 32 classes were considered.

We have applied transfer learning on deep-learning models that are being used successfully for object recognition [25], notably the Inception-Resnet-v2 [26] and the Resnet-101 [18] models. In this way, we were able to achieve high levels of efficiency while minimizing the amount of data required for training, if compared with a random weight initialization approach. Initial models were chosen trying to maximize the overlap of classes already considered in these models and classes aimed in the final model. One model for each specific scenario was considered. A dedicated dataset of 1500 images, containing a total of 10733 objects belonging to the classes defined for the fashion use case, was manually annotated, creating the ground truth for training and evaluating the models. A similar approach was adopted for the other application scenarios.

**Figure 2:**
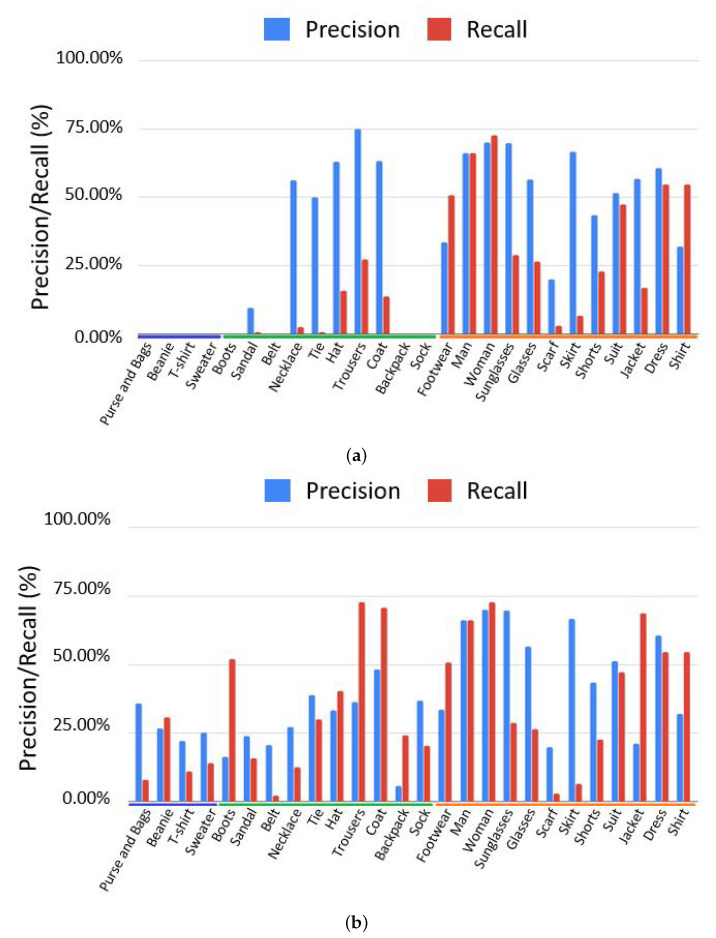
Faster-RCNN Inception-Resnet v2. (**a**) Before Transfer Learning. (**b**) After Transfer Learning.

**Figure 3:**
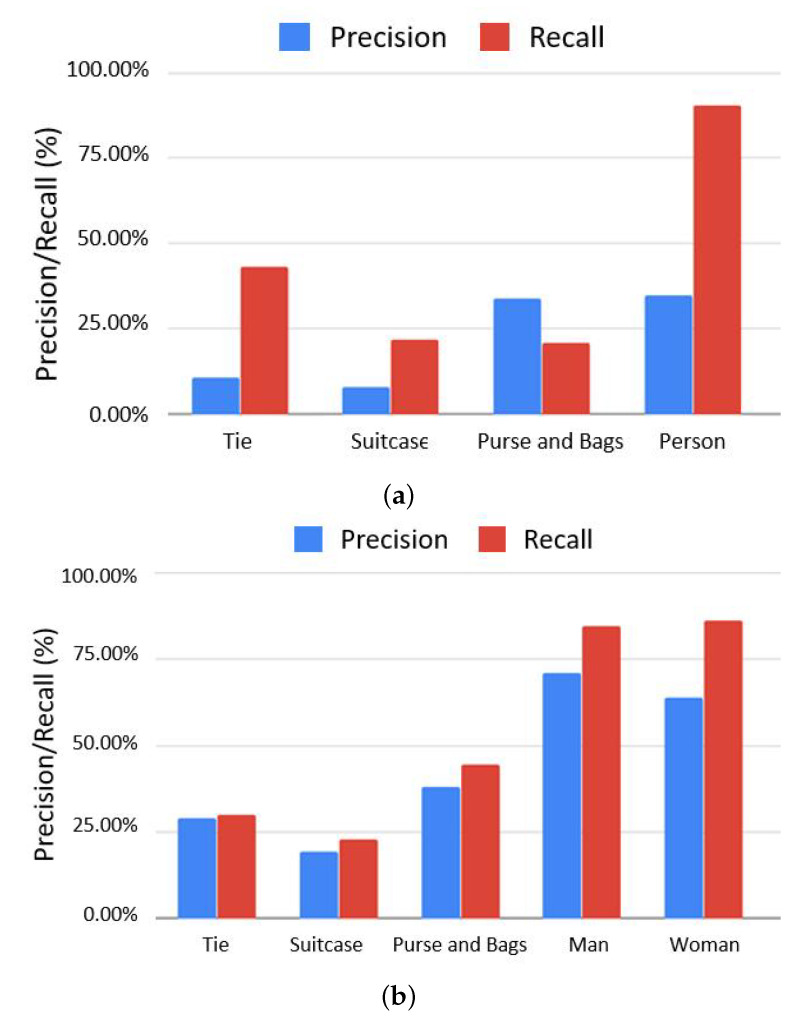
Faster-RCNN Resnet-101. (**a**) Before Transfer Learning. (**b**) After Transfer Learning.

Figure 2 presents a comparison of the results obtained before and after having re-trained the Inception-Resnet-v2 model with our dataset (which had been pre-trained with the Open-Images-v4 dataset [27]). Figure 3 shows the results of a similar testing procedure using the Resnet-101 model (which had been pre-trained with the COCO dataset [28]). The list of classes in each of the models can be extended (i.e., adding data for new classes) or enhanced to increase the accuracy (i.e., adding more images to the existing classes).

While analyzing the results obtained with the Inception-Resnet-v2 model, we have realized that they could be interpreted by separating them in three clusters. These clusters are highlighted with different colors along the horizontal axis of Figure 2. The blue cluster gathers new classes of objects that were added to the model in our work (“Beanie”, “Purse and Bags”, “T-shirt”, “Sweater”). The green cluster refers to classes that were already contemplated by the baseline model and for which the recall was improved. Finally the yellow clusters represents the already existing classes for which the model performance did not improve. Such results demonstrated that it would be feasible to use existing models for detecting new concepts not previously contemplated, among which are those that were essential for the implementation of the intended automatic creative process in FiM. The drop in precision that can be noticed for some items on the green cluster can be explained by the introduction of new classes representing visually similar objects (e.g., “Hat” and “Beanie”, “Coat” and “Sweater”), increasing the inter-class noise. However, it is unlikely that mixing very alike objects will have a negative impact on storytelling purposes. Moreover, in the cases where precision has decreased, we still registered an overall positive evaluation, given the significant improvement observed on the recall metric. In fact, the baseline model was able to detect only an extremely small number of such objects compared with the number that effectively existed in the images. Such were the cases of the classes “Necklace” and “Tie”, which were rarely detected. So, even with a noticeable decrease in precision, the significant improvement on recall is still a positive outcome.

Results obtained with Resnet-101 (Figure 3) also demonstrated that performance could be improved for existing classes. Likewise, that new concepts could be efficiently detected, as is the case for the person’s genre class. Given the limited number of classes available in the baseline model, the inter-class confusion observed with the Inception model was not noticeable, enabling to enhance both recall and precision for most cases.

**Figure 4:**
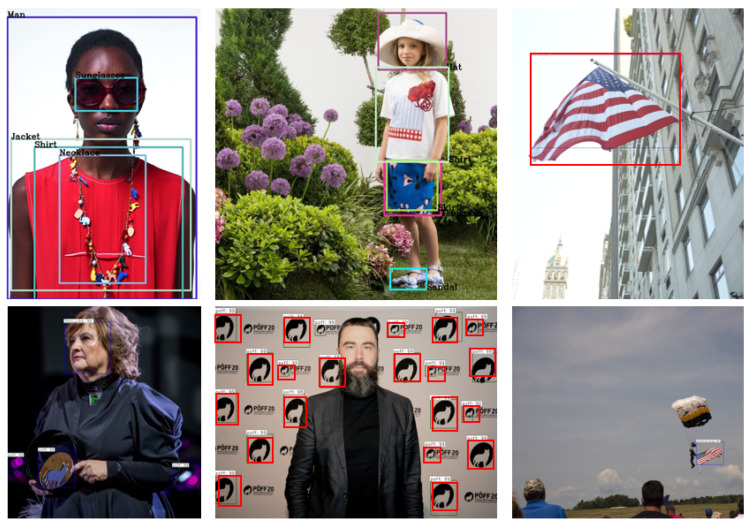
Example of object detection in the different application scenarios.

Figure 4 shows some results obtained using the re-trained models for recognizing fashion items, symbols (more specifically, flags and logos) and iconic personalities, which are all types of objects relevant for the FiM defined scenarios.

**Figure 5:**
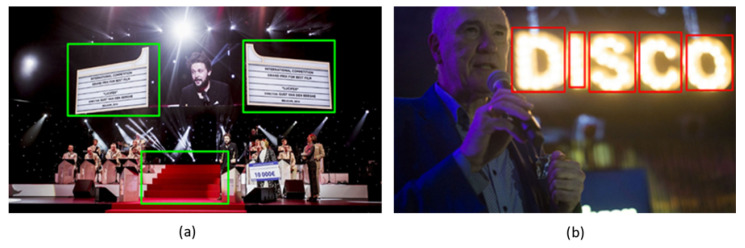
Relevant low-level regions. (**a**) Red Carpet, Screens (**b**) Spotlights.

Despite the robust performance of deep learning methods, the possible disparity of contents present in photographs can naturally lead to the deep learning models not being able to detect objects of any of the defined classes. To cope with these situations and guarantee that region information is passed to the subsequent modules, we have developed computer vision algorithms that operate at the pixel level and are use-case agnostic. The works proposed in [29,30] describe interesting approaches for the accurate and efficient segmentation of objects in video, taking advantage of motion and temporal information. However, in the current proposal, we deal with single still-shot images, which does not allow the applicability of these proposals. Our solution extracts luminance features to detect regions whose pixels have luminance values in the top tier of the luminance range of the image. Color is also used to segment regions with larger difference with regards to the overall image using the CIEDE200 metric [31,32]. This enables the detection of interesting regions with an undefined shape, such as fire, explosions, stages or spotlights. Some examples are illustrated in Figure 5.

### 4.3. Assisted Annotation Tool

**Figure 6:**
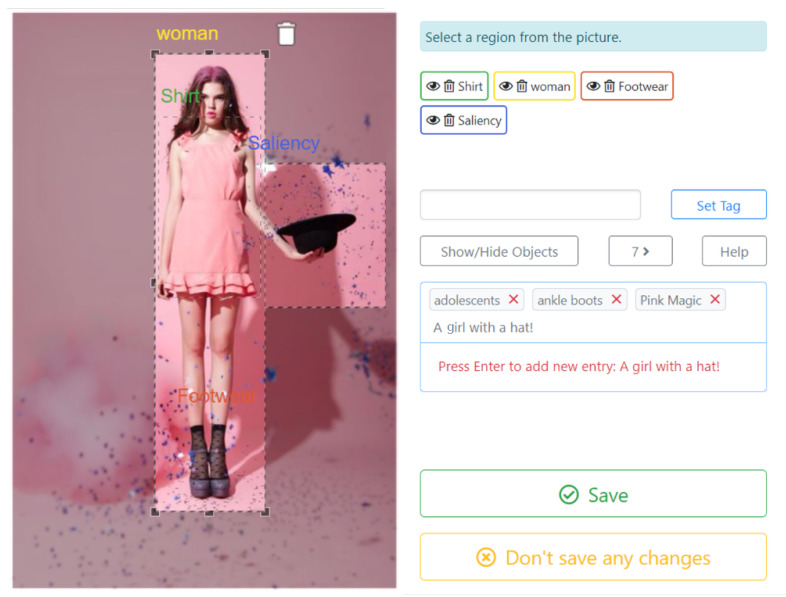
Assisted annotation tool.

Metadata can also be obtained through a collaborative and assisted process in which users can improve/correct the automatically generated annotations or even define additional RoIs. In this process, the user may select keywords from a pre-defined list (matching the defined classes or concepts) or create generic tags that might contribute to enhance the creative workflow (e.g., by providing text to be included as captions). While enabling this enrichment of annotations, the Assisted Annotation Tool (AAT) can also be used to generate new ground-truth datasets, either to improve the performance of the Neural Network (NN) models or to fit them to new scenarios. Figure 6 depicts a screenshot of the AAT illustrating the identification of RoIs and labels.

## 5. Semantically Aware Storytelling

The final step of the workflow is the automatic creation of an engaging video clip, having as a starting point the 2D static footage, consequently enriching it so that diversified and dynamic multimedia content is obtained. This is achieved by applying filters, 2D, 3D and motion effects to the image or on a region basis, made possible by the availability of the previously generated metadata.

Filters are operations that modify characteristics of static content without animating it. Traditional filters such as brightness or color modification can be applied either to the full image or on an RoI basis.

On the other hand, effects are dynamic modifications applied to the media, changing over time, with the purpose of narration. In 2D traditional effects, motion is limited to rotations and translations in a 2D plane, e.g., the pan-effect that moves the viewpoint from left to right over the image or a given RoI, the rotations of objects, or zooming in and out of RoIs.

**Figure 7:**
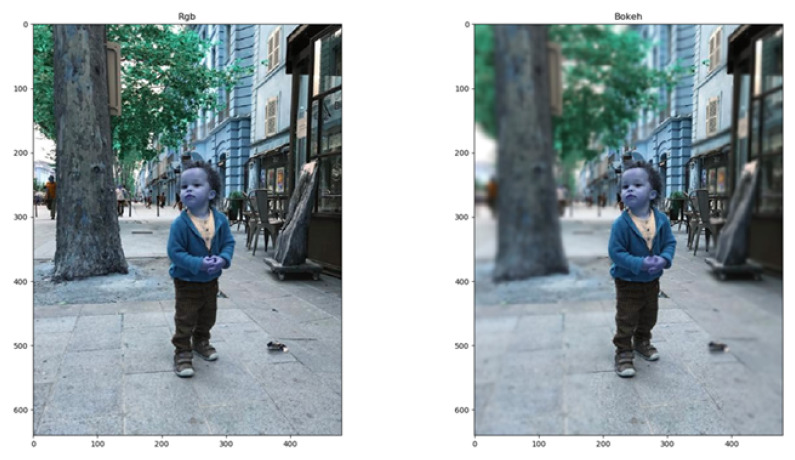
Bokeh effect applied to an identified RoI.

**Figure 8:**
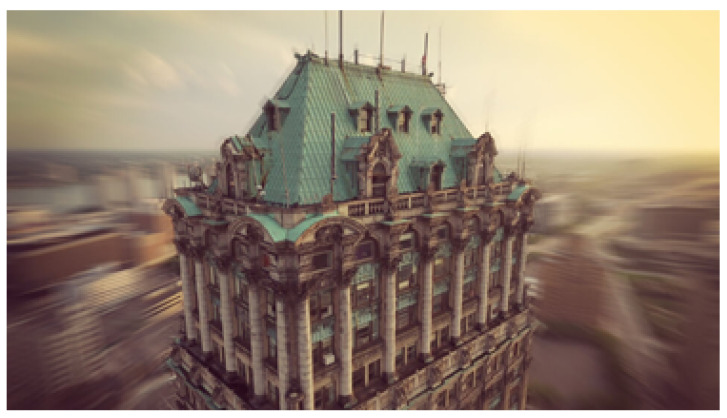
Vertigo effect applied to an identified RoI.

To apply wider ranges of motion effects, specified in a 3D space, depth information is extracted from the original photograph. The depth estimation algorithm is based on the principles of “Shape-from-Shading” and “Depth-from-Luminance” to estimate depth for identified dominant frequency bands. These bands are then merged to assemble a depth image, by using fixed weight factors, that can be used in a CNN trained with various types of photographs. The defined 3D effects include motion-blur, bokeh and vertigo effects. When introducing an artificial motion blur in the photo, the focus range is related to the depth of the object or RoI in the scene. The artificial bokeh effect, illustrated in Figure 7, puts the main RoI of an image in focus with a shallow depth of field so that the background will be strongly blurred. By leaving only the subject sharp, the viewer’s attention is drawn to this specific region. The vertigo effect is implemented by moving the film camera on a dolly away from or towards the object while zooming in and out, as illustrated in Figure 8.

The core of the storytelling automation is metadata, and the narration engine is the central piece in this process of using metadata to generate semantic-aware video stories. Different levels of automation are offered in the creative process, enabling the customization of the solution to different types of expertise on editing content in the creative process.

Full automatic storytelling can be achieved through support from the identification of RoIs, associated labels, and sizes and positions in the image, as well as by the situational information collected at the moment of shooting. A set of knowledge-based rules were implemented, depending on the application scenarios to decide on the relevance that is given to each object in the storytelling process (e.g., Where in the photo should the animation start or end? How long should an object appear in the timeline? What effect will improve the visual experience?). Another case-oriented rule was implemented to identify the number of objects of the same class that have been detected, e.g., having several jewelry objects identified may imply that this is a jewelry fashion show, and motion can be generated so that a timeline focusing on this class of objects is created. Aside from these object-based rules, environmental context metadata enables inferring additional information and refining the automatic production. Additional rules can be added to the platform to cope with other application scenarios or creative options.

**Figure 9:**
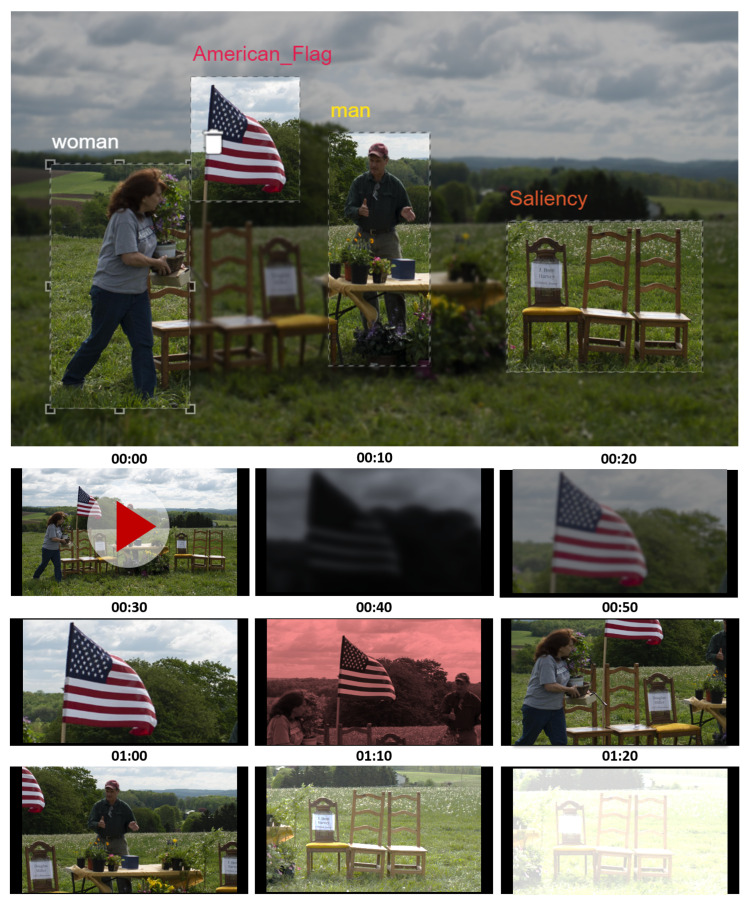
Representation of a time sequence, as an illustration of the final animation, taking a single image as baseline.

Additionally, the concept of templates was implemented, enabling users to pre-define settings to be used for storytelling purposes. Creative personnel can decide the customization of several parameters that will be used to render the video: a director may have a preference for a zoom filter while another may have a preference for a pan-and-tilt effect; one may want a style where movement is fast while another may opt for slow-motion, etc. Additionally, for instance, for a zoom-pan template, the order to navigate from item to item can be configured, together with a movement speed and a rest period per item. The information on item locations, sizes and labels previously acquired is then used to automatically create the intended animation. An illustration of an automatically generated clip, with animations based on acquired metadata, is presented in Figure 9.

## 6. Discussion and Conclusions

Three distinct pilots were organized to allow for testing the developed system in real-world conditions and validating the concept and its usefulness for the media and creative industries. The pilots were defined according to the three use-case scenarios addressed by the project, namely, photojournalism/media, fashion/marketing and festivals/events. To enable gathering coherent and relevant data from the three pilots, covering the different functionalities offered by the system while creating the conditions for the pilot testers to perform their role in an easy, efficient and manageable way, guidelines describing the tasks the user should execute with the tool were elaborated and a common methodology was defined for collecting their feedback. It was decided to do it using a concise questionnaire, covering the following eight performance and usability indicators:

1. User Interface Design
2. Speed and Accuracy
3. Object Identification
4. 3D Effects
5. Templates
6. Automatic Storytelling Features
7. Market Readiness
8. Output Quality

For all of these aspects, positive feedback was obtained from at least 75% of the pilot testers. A total of 78% were impressed with the accuracy of the annotations. Overall, this parameter received an average score of 4+ on a scale from 1 (lowest) to 5 (higher). The majority considered that the tool produced decent automatic stories with good-quality effects, serving very well the intended purpose of on-the-spot dissemination in appealing ways.

The possibility of resorting to templates to control the generation of the multimedia story in an easy way was also acknowledged. The testers also appreciated the speed of the tool and reported less than 20 s needed for objects to be identified, 10–30 s needed for defining a new story using the template approach, 60–90 s when not using any template and 30–60 s for the automatic storytelling tool to create a video. Table 1 summarizes some of the most relevant user feedback.

Overall, users stated that the tool would have a positive impact on their activities, and it was classified as a value-added solution, namely in shortening production time and in creating relevant semantic-aware animations automatically. Additionally, it was heavily emphasized that it creates an opportunity for re-purposing archive images, especially when it comes to social media campaigns. Users also commented that it was great to take a photo and effortlessly publish in less than one minute a video clip conveying the desired message on social media.

Even though appealing and promising results have already been obtained, there is still room for improvement, notably at the research level, not only concerning the performance of the NN models but also to go one step forward and enable the identification of higher-level concepts in the photograph. We believe this can be achieved by combining different approaches for extracting metadata and fusing such metadata. In this context, we intend to further explore the use of case-oriented rules and reasoning. Another area of study and experimentation concerns the reduction in the sizes of datasets needed to re-train models, aiming at the incorporation of new concepts.

## Figures and Tables

**Table 1:** Evaluation summary.

Qualitative Evaluation	
Overall positive feedback on the 8 evaluation items (score: 3–5)	75%
Excellency of the annotations (score: 4–5)	78%
Average scoring of the annotations	4+
**Operation**	
Time required for object identification	20 s
Time required to customize a new story, template-based	10–30 s
Time required to customize a new story without using templates	60–90 s
Time required for the system to render a video	30–60 s

## Data Availability

Not applicable.
